# Effectiveness and sustainability of a structured group-based educational program (MEDIHEALTH) in improving medication adherence among Malay patients with underlying type 2 diabetes mellitus in Sarawak State of Malaysia: study protocol of a randomized controlled trial

**DOI:** 10.1186/s13063-018-2649-9

**Published:** 2018-06-05

**Authors:** Chuo Yew Ting, Shahren Ahmad Zaidi Adruce, Mohamed Azmi Hassali, Hiram Ting, Chien Joo Lim, Rachel Sing-Kiat Ting, Abu Hassan Alshaari Abd Jabar, Nor Anizah Osman, Izzul Syazwan Shuib, Shing Chyi Loo, Sui Theng Sim, Su Ee Lim, Donald E. Morisky

**Affiliations:** 1 Institute of Borneo Studies, Universiti Malaysia Sarawak, Kota Samarahan, Sarawak Malaysia; 2 Discipline of Social and Administrative Pharmacy, School of Pharmaceutical Sciences, Universiti Sains Malaysia, Penang, Malaysia; 3 Sarawak Research Society, Sarawak, Malaysia; 4 Clinical Research Center, Sarawak General Hospital, Sarawak, Malaysia; 5 Jeffrey Cheah School of Medicine and Health Sciences, Monash University Malaysia, Selangor, Malaysia; 6 Pharmaceutical Services Division, Sarawak State Health Department, Sarawak, Malaysia; 7 Pharmacy Practice and Development Division, Sarawak State Health Department, Sarawak, Malaysia; 8 Pharmacy Enforcement Division, Sarawak State Health Department, Sarawak, Malaysia; 9 Department of Community Health Sciences, UCLA Fielding School of Public Health, Los Angeles, CA USA

**Keywords:** Culturally sensitive, Type 2 diabetes mellitus, Medication adherence, Malay patients, Group-based educational program, Randomized controlled trial

## Abstract

**Background:**

Amidst the high disease burden, non-adherence to medications among patients with type 2 diabetes mellitus (T2DM) has been reported to be common and devastating. Sarawak Pharmaceutical Services Division has formulated a pharmacist-led, multiple-theoretical-grounding, culturally sensitive and structured group-based program, namely “Know Your Medicine – Take if for Health” (MEDIHEALTH), to improve medication adherence among Malay patients with T2DM. However, to date, little is known about the effectiveness and sustainability of the Program.

**Methods/design:**

This is a prospective, parallel-design, two-treatment-group randomized controlled trial to evaluate the effectiveness and sustainability of MEDIHEALTH in improving medication adherence. Malay patients who have underlying T2DM, who obtain medication therapy at Petra Jaya Health Clinic and Kota Samarahan Health Clinic, and who have a moderate to low adherence level (8-item Morisky Medication Adherence Scale, Malaysian specific, score <6) were randomly assigned to the treatment group (MEDIHEALTH) or the control group. The primary outcome of this study is medication adherence level at baseline and 1, 3, 6 and 12 months post-intervention. The secondary outcomes are attitude, subjective norms, perceived behavioural control, intention and knowledge related to medication adherence measured at baseline and 1, 6 and 12 months post-intervention. The effectiveness and sustainability of the Program will be triangulated by findings from semi-structured interviews with five selected participants conducted 1 month after the intervention and in-depth interviews with two main facilitators and two managerial officers in charge of the Program 12 months after the intervention. Statistical analyses of quantitative data were conducted using SPSS version 22 and Stata version 14. Thematic analysis for qualitative data were conducted with the assistance of ATLAS.ti 8.

**Discussion:**

This study provides evidence on the effectiveness and sustainability of a structured group-based educational program that employs multiple theoretical grounding and a culturally sensitive approach in promoting medication adherence among Malays with underlying T2DM. Both the quantitative and qualitative findings of this study could assist in the future development of the Program.

**Trial registration:**

National Medical Research Register, NMRR-17-925-35875 (IIR). Registered on 19 May 2017. ClinicalTrials.gov, NCT03228706. Registered on 25 July 2017.

**Electronic supplementary material:**

The online version of this article (10.1186/s13063-018-2649-9) contains supplementary material, which is available to authorized users.

## Background

Diabetes is an epidemic chronic disease caused either by the failure of the pancreas in producing sufficient insulin or by the failure of the body cells in utilizing the insulin produced by the pancreas effectively [1]. The majority (95%) of patients with diabetes have type 2 diabetes mellitus (T2DM), which is more common in adults than in children [1, 2]. A national health and morbidity survey (NHMS) carried out by the Ministry of Health, Malaysia (MOH), in 2015 revealed that 17.5% (95% Confidence interval 16.6, 18.3) of Malaysians were found to have diabetes [3]. In comparison with the results of the first NHMS and the second NHMS conducted in 1986 and 1996, respectively, the recent findings again showed that there is a steady increase in the prevalence of diabetes among Malaysians [4]. Besides, the national survey found that there is a significant difference between different ethnic groups. Indians were found to have the highest prevalence of diabetes (22.1%, 95 CI 19.2-25.3), followed by the Malays (14.6%, 95 CI 13.8-15.5), the Chinese (12.0%, 95 CI 10.7-13.5) and last the other indigenous groups (10.7%, 95 CI 8.8-13.0). Even though Indians were found to have a greater percentage of T2DM than Malays, Malays have a higher total number of T2DM patients owing to their bigger population size in Malaysia than Indians, which is the third largest ethnic group in Malaysia [5].

Amidst the high disease burden, non-adherence to medications among patients with T2DM has been reported to be common and devastating [6]. It has been estimated that more than 50% patients fail to achieve recommended glycaemic goals due to non-adherence to diabetic medications [7, 8]. There is no exception for Malaysia as a recent national survey revealed that 73.1% of Malaysians who are on medication did not adhere to the medications prescribed [9]. 

A recent systematic review [10] summarized that the factors that contribute to poor adherence among T2DM patients are age, ethnicity, health beliefs, medication cost, insulin use, health literacy, medication cost, co-pays, medical insurance coverage and primary non-adherence. Furthermore, on the one hand, a greater adherence rate was significantly associated with better glycaemic control, less hospital visits and admissions, and lower medical costs. On the other hand, a lower adherence rate was significantly associated with poor medication tolerance, frequency of medication intake (more than two times a day), having concomitant depression and negative belief about the medications. Consequently, patients who poorly adhere to medications take more medications owing to poor glycaemic control and the development of micro- and macrovascular complications [11]. Furthermore, the condition further worsens their adherence owing to more complex medications and more side effects experienced [7]. This inevitably increases the financial burden and wastage to health services [12]. Hence, breaking the vicious cycle should be an urgent priority for all stakeholders.

In Malaysia, the Pharmaceutical Services Division (PSD) of the MOH launched in 2007 a campaign called “Know Your Medicine” (KYM) to promote the quality use of medicines [13]. The campaign utilizes mass media, social media and a group-based educational program (GBEP) to deliver the message to the Malaysian public. The messages conveyed include information on their medication management, such as why, how and when to take medicines, reporting adverse drug events, awareness on the rational use of medicines and medications that need special precautions. In particular, assuring and improving medication adherence among patients is one of the important messages conveyed through the campaign. Moreover, the PSD of Sarawak State Health Department further expanded the scope of the GBEP by formulating a 3-h pharmacist-led culturally sensitive structured GBEP in promoting medication adherence among T2DM patients [14]. The official name of the structured GBEP is “Know Your Medicine – Take it for Health” (MEDIHEALTH). Notably, MEDIHEALTH is a culturally sensitive and culturally appropriate program that is tailor-made to suit the cultural differences of the major ethnicities in the State, including Iban, Bidayuh, Malay and Chinese. In addition, MEDIHEALTH was specifically designed to complement the individual approach in improving medication adherence among T2DM patients called Diabetes Medication Therapy Adherence Clinic (DMTAC) initiated by the PSD, MOH [15]. Previous literature supports the employment of a structured GBEP such as MEDIHEALTH because it has numerous irreplaceable benefits, including (1) validation, (2) normalization of experience, (3) reduction of isolation, (4) sense of belonging and (5) enhanced self-esteem [16]. The design and content of MEDIHEALTH will be further elaborated in the “Methods/design” section.

While the previous literature has shown that a GBEP in promoting self-management among T2DM patients has proven to be effective [17], little has been done to examine the effectiveness of a structured GBEP in improving medication adherence among T2DM patients [18]. Furthermore, we echo what has been mentioned by Edmondson and colleagues in that “very few” studies of behaviour change interventions had actually tested the mechanism of behavioural interventions [19]. Such a gap in the knowledge has caused researchers to remain unclear on why and how an intervention works or fails and thus has limited future intervention development [19]. Hence, by investigating the effectiveness of the structured GBEP MEDIHEALTH and its mechanism, this study intends to provide managerial implications to the PSD, Malaysia, and to bridge the gap in the extant body of knowledge.

Apart from focusing on the effectiveness of MEDIHEALTH, its sustainability is also an important component to the organizations that formulate, implement and finance the program [20, 21]. Moreover, health interventions that are not sustainable will result in participants becoming disillusioned and hinder participants from future engagement in any health interventions that could be beneficial to them [21]. Thus, this study will also evaluate the sustainability of the program since it is still at the early phase of implementation. Notably, researchers employ the perspectives of Pluye and colleagues in conceptualizing sustainability, which involves organizational routines and institutional standards [22]. Moreover, we employ a qualitative approach to evaluate the sustainability of the program “to understand the phenomenon, refine hypotheses, and develop strategies to promote sustainment”, as recommended by Stirman and colleagues [23].

In terms of theoretical grounding to evaluate the change in medication-adhering behaviour, on the one hand, the theory of planned behaviour (TPB) was chosen as the underlying theory in evaluating the effectiveness of MEDIHEALTH as it has the strongest empirical evidence in medication adherence studies [24]. On the other hand, the information-motivation-behavioural skills model (IMB) was chosen as the supporting theory as it has been empirically tested on medication adherence among T2DM patients [25].

On the one hand, the TPB is a theory founded in the field of social psychology and is best suited to describe volitional behaviours, especially health-related behaviours [26]. It posits that a behaviour is influenced by attitude towards the behaviour, perceived subjective social norms towards the behaviour and perceived behavioural control towards the behaviour and is mediated by the intention to act. Attitude towards the behaviour is how one evaluates the advantages or disadvantages of performing a behaviour. Subjective social norm is the social expectations of a behaviour perceived by an individual. Perceived behavioural control is personal perception about the difficulty and capability of performing a behaviour. Ajzen further revealed that perceived behavioural control has a direct effect on the behaviour when a given behaviour has less volitional control. Perceived behaviour control could reflect both external (such as time and money) and internal (such as skill and information) factors, which is similar to the self-efficacy construct conceptualized by Bandura [27]. Thus, the level of perceived behavioural control of an individual will determine the persistence of a given behaviour over time in the face of obstacles and setbacks. For a better illustration of the value of the TPB in explaining behaviour in this study, medication-taking behaviour (medication adherence) is influenced by attitude, perceived social norm and perceived behavioural control over medication adherence, and all three factors are mediated by intention towards medication adherence, whereas perceived behavioural control has also a direct effect on the behaviour. A recent systematic review and meta-analysis on the use of TPB in the studies of adherence to treatment in chronic illness further supports the feasibility of the TPB in the current study [24]. The TPB was found to account for 32.92% of the variance in intention and 9.18% of the variance in behaviour, and all the relationships between variables were found to be consistent with the original hypotheses of the TPB.

On the other hand, the IMB is a simple and the latest behavioural model that has a high predictive value on long-term medication adherence [28]. It had been studied among patients affected by tuberculosis [28], HIV [29] and T2DM [25], who are required to adhere to long-term medication to achieve a good clinical outcome. The IMB posits that to achieve behavioural change, one should have adequate behaviour-related information; health behaviour motivation, which consists of personal motivation and subjective motivation; and necessary skills to perform a specific health behaviour [29]. Notably, there are similarities between IMB variables and TPB variables. Firstly, the motivation constructs of the IMB, which consist of personal motivation and social motivation, are like the attitude and subjective norm of the TPB, respectively. Secondly, the behavioural skill variable is like perceived behavioural control. In terms of the relationship between the three independent variables, the behavioural skill variable serves as a mediator that mediates the effect of behaviour-related information and health behaviour motivation towards the health behaviour. However, both behaviour-related information and health behaviour motivation have a direct effect on health behaviour.

**Fig. 1:**
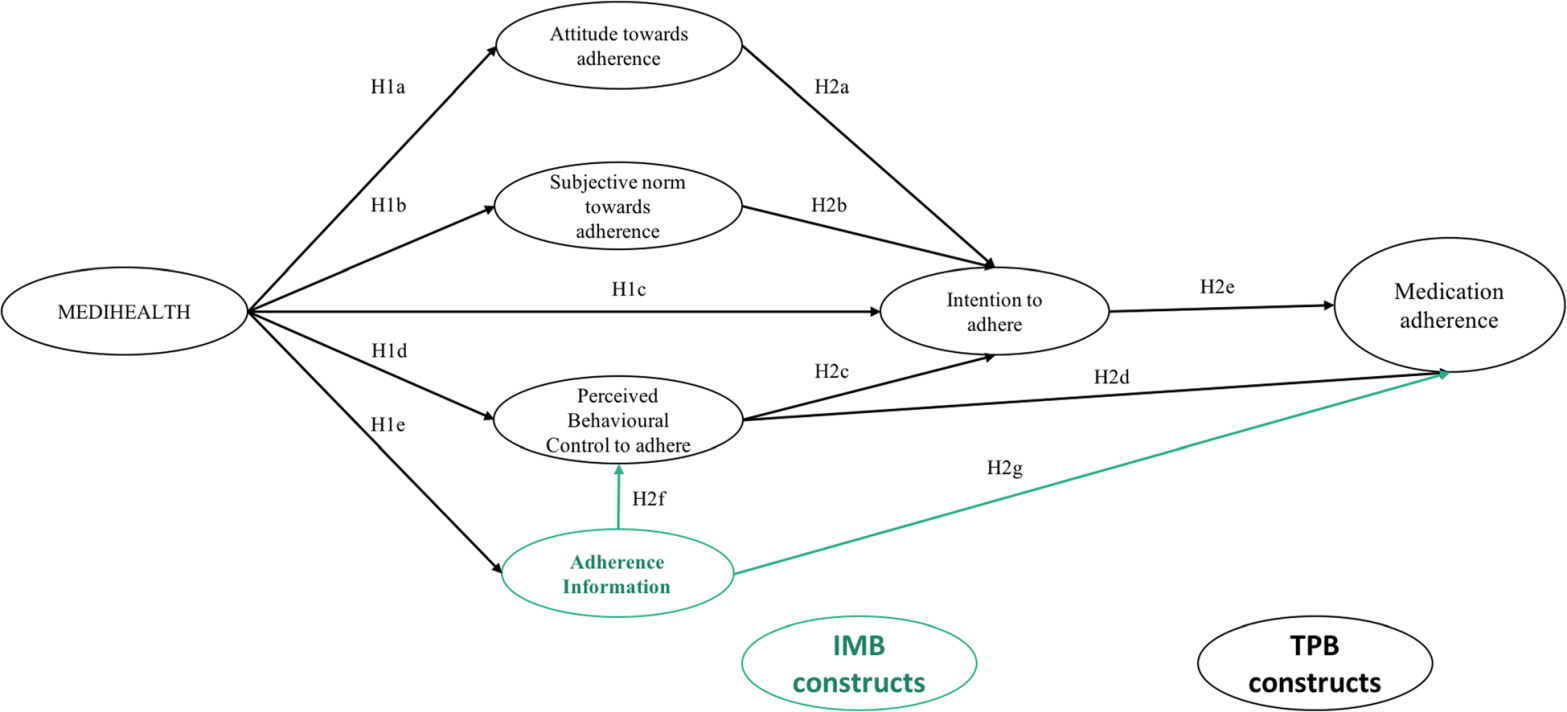
Conceptual framework of the study (TPB constructs are in black while IMB construct is in green)

By comparing the constructs of the IMB and TPB in explaining a behaviour, regardless of the relationship between constructs, the main difference is that the IMB has an additional construct, which is behaviour-related information. In the IMB, the behaviour-related information construct has a direct effect on health behaviour and behavioural skills. By noting the similarity of both theories, the researchers hypothesize that the behaviour-related information construct of the IMB serves as an independent variable which has a direct effect on the perceived behavioural control construct and the medication adherence construct of the TPB. Such a hypothesis is aimed at increasing the explanatory power of the underlying theory towards medication-taking behaviour through extending the TPB. Nevertheless, MEDIHEALTH is included as one of the independent variables that will improve the scoring of extended TPB variables. The conceptual framework of this study is presented in Fig. 1.

This study aims to investigate the effectiveness of MEDIHEALTH in improving medication adherence among Malay communities with underlying T2DM in the Sarawak State of Malaysia. The specific objectives of this study are (1) to measure the effectiveness of MEDIHEALTH in improving the medication adherence level and the component of the extended TPB, (2) to identify the component of the extended TPB that predicts medication adherence after participating in MEDIHEALTH, and (3) to investigate the sustainability of the program. Based on the first specific objective, eight hypotheses are to be tested:

- H1a: Increase in intention to adhere would be mediated by improvements in attitude towards adherence after baseline, which is achieved by participating in MEDIHEALTH.
- H1b: Increase in intention to adhere would be mediated by improvements in subjective norm towards adherence after baseline, which is achieved by participating in MEDIHEALTH.
- H1c: Increase in medication adherence would be mediated by improvements in intention to adhere after baseline, which is achieved by participating in MEDIHEALTH.
- H1d: Increase in intention to adhere would be mediated by improvements in perceived behavioural control towards adherence after baseline, which is achieved by participating in MEDIHEALTH.
- H1e: Increase in intention to adhere would be mediated by improvements in adherence information after baseline, which is achieved by participating in MEDIHEALTH.
- H1f: Before the intervention, there are no significant differences of medication adherence level and the psychosocial variables related to it among the participants between the intervention group and the control group.
- H1g: After 1, 3, 6 and 12 months of the program, the medication adherence levels among the participants in the intervention group are significantly greater than the medication adherence levels before the intervention.
- H1h: After 1, 3, 6 and 12 months of the program, the medication adherence levels among the participants in the intervention group are significantly greater than the medication adherence levels of the participants in the control group.

For the second specific objective, seven hypotheses are to be tested:

- H2a: Improvement in attitude towards adherence will contribute to the increase in intention to adhere.
- H2b: Improvement in subjective norm towards adherence will contribute to the increase in intention to adhere.
- H2c: Improvement in perceived behavioural control towards adherence will contribute to the increase in intention to adhere.
- H2d: Improvement in perceived behavioural control towards adherence will contribute to the increase in medication adherence.
- H2e: Improvement in intention to adhere will contribute to the increase in medication adherence.
- H2f: Improvement in adherence information will contribute to the increase in perceived behavioural control towards adherence.
- H2g: Improvement in adherence information will contribute to the increase in medication adherence.

## Methods/design

### Study design

**Fig. 2:**
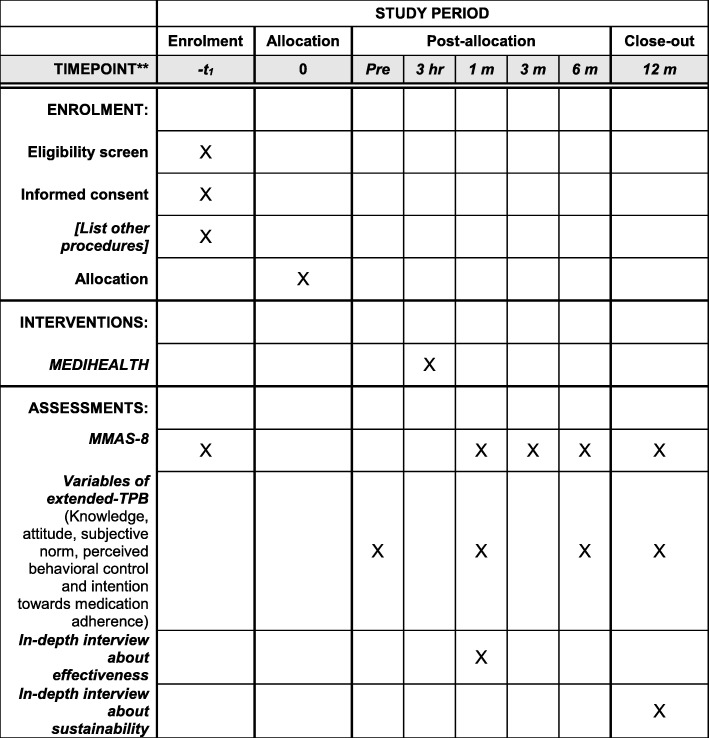
SPIRIT figure. Schedule of enrolment, interventions and assessments. *MMAS-8* 8-item Morisky Medication Adherence Scale, Malaysian specific; *TPB* theory of planned behaviour

Given the nature of the research problem, an experimental study design will be utilized to examine the effectiveness of the structured GBEP MEDIHEALTH. In particular, it was a prospective, multicentre and parallel-design randomized controlled with two treatment groups. The protocol is written in accordance with the Standard Protocol Items: Recommendations for Interventional Trials (SPIRIT) checklist (Additional file 1), and its figures are illustrated in Fig. 2. This trial protocol is registered with ClinicalTrials.gov (NCT03228706).

### Study population and setting

As mentioned above, Malays have the greatest number of patients with T2DM in the nation. Thus, the researchers made an informed decision by selecting Malay patients with underlying T2DM as the studied population for the current study. Moreover, since MEDIHEALTH is a structured GBEP formulated by the Sarawak State of Malaysia and has only been implemented in the State, the studied population will be entirely Malay patients with underlying T2DM who obtain their medications in Sarawak during the period of this study. Samples were selected from the Malay communities who reside at Kuching North City Hall and Samarahan Division of Sarawak State, which have the largest proportion of Malay communities residing in the State [5]. The majority of Malay communities are able to access quality healthcare services provided by the Petra Jaya Health Clinic (PJHC) and the Kota Samarahan Health Clinic (KSHC). Notably, Malays who are diagnosed with T2DM receive continuous consultation and monitoring at the clinics. Hence, with the aim of recruiting suitable respondents that could yield highly generalizable data to the studied population, PJHC and KSHC have breen chosen as the study sites to recruit respondents and collect data. Moreover, PJHC and KSHC are to be included as the KYM campaign sites. Thus, the researchers incorporated this study into the KYM campaign by obtaining prior approval from Sarawak Pharmaceutical Services Division.

### Participants

#### Sample size

The current study is the first to investigate the effectiveness and sustainability of the program since the program was developed in late 2016 and began to test run in early 2017. Hence, we aim to achieve such objectives with minimum resources and time required before this program is expanded to other Malay communities in Malaysia. This is a study of a continuous response variable from independent control and experimental subjects where one control per experimental subject is planned. In a previous study [30], the response within each subject group was normally distributed with a standard deviation of 1.8. If the true difference in the experimental and control means is 1, we need to study 69 experimental subjects and 69 control subjects to be able to reject the null hypothesis that the population means of the experimental and control groups are equal with a probability (power) of 0.9 [31]. By estimating 30% of dropout or incomplete data, a minimum sample size of 180 with 90 for both groups is pre-determined.

#### Selection criteria

The inclusion criteria for the participants are (1) 8-item Morisky Medication Adherence Scale (MMAS-8), Malaysian specific, score <6 and (2) Malay T2DM patients; the exclusion criteria are (1) pregnant women, (2) patients below 18 years old, (3) patients who have severe and enduring mental health problems, (4) patients who cannot listen or read owing to inherited disabilities or malfunction, (5) patients who are unable to communicate in the Malay language, (6) patients who are participating in other studies, (7) patients who decline consent to participate and (8) hospitalized patients.

#### Confounding variables

Based on evidence from previous literature [32, 33], the confounding variables included in this study are (1) route of administration (oral only or oral and insulin injection); (2) number of medications (one or more than one); (3) frequency of medications (a once-daily dose or more frequent); (4) age; (5) sex; (6) highest education level; (7) monthly household income; (8) employment status; (9) having complications; (10) taking traditional complementary and alternative medicines; (11) residential area (urban or rural); (12) living conditions (having social support or living alone); (13) having received diabetic education by a diabetic nurse and (14) enrollment with DMTAC.

#### Recruitment

Prospective and eligible respondents will be recruited consecutively from the two selected health clinics during their routine scheduled visits before intervention. The MMAS-8, Malaysian specific, which has been validated among Malaysians with T2DM [34, 35], will be administered to obtain the adherence score, enabling us to evaluate the eligibility of the prospective respondents. Patients who have a low adherence (MMAS-8 score <6) and fulfil all the selection criteria will be provided with a study information sheet and informed consent. Those who agree to participate will be informed about the program, and asked to choose a date to attend the program. After they choose their preferred date, their name will be recorded in the “List of Participants” with a specific code assigned. Notably, patients will not be informed of their group assignment and thus will not be not aware of the differences between the intervention group and the control group. Besides, none of the recruiters, who are pharmacist staff at both health clinics, is aware of subsequent treatment allocation throughout the recruitment stage, to ensure allocation concealment. All research materials that contain patients’ information will be coded and kept by the principal investigator to maintain the confidentiality of respondents.

#### Randomization and blinding

The Sarawak Research Society, which is independent from the study team, will be appointed to carry out simple randomization. The List of Participants will be handed over to the chairman of the Society to carry out the randomization. The randomization will be conducted using an online randomization program available at http://www.graphpad.com/quickcalcs/index.cfm, as recommended by Suresh [36]. After that, the participant list, with their code and group assignment, will be kept by the chairman of the Society without informing any of the researchers, facilitators or respondents, to ensure the blinding of the three parties to treatment allocation. On the day of the intervention, the chairman and the authorized committee member of the society will register the attendance of respondents and inform them about the actual venue of the program. PJHC and KSHC will have two venues each prepared and available during the period of the study, with one venue allocated to the intervention group and the other one allocated to the control group. None of the participants will be aware of the difference between the venues assigned to them, whereas the facilitators will be. However, participants will know whether they are assigned to the control group as there will not be any form of information provided to the participants in this group. Hence, the blinding of participants and facilitators will end at the intervention stage. The blinding of the researchers will be continued towards the publication stage. During the post-intervention follow-up, the researchers will still be blinded to the allocation of participants, as the participant list with group allocation will be kept by the chairman of the society.

### Intervention design

#### Intervention mapping

As mentioned before, the structured GBEP MEDIHEALTH employed both psychosocial and behavioural approaches, with multiple behavioural theories as theoretical grounding. It was mapped based on the application of behaviour change theories as recommended by Slater [37]. Moreover, it is a culturally sensitive structured GBEP that employs three components of culturally sensitive intervention, including bilingual facilitators and materials, literacy-appropriate materials and social support [38]. The intervention mapping and content of the intervention is available in Additional file 2. Notably, this stage-of-change framework, which incorporates the social learning theory, elaboration likelihood model, theory of planned behaviour, self-efficacy theory and information-motivation-behavioural skills model, was used to map the program but does not serve as an empirical measure of the mechanism. The empirical measures of the mechanism in our study are the variables of the extended TPB, which integrates the TPB and IMB, as aforementioned.

In comparison with the six-step model in quality intervention development proposed by Wight and colleagues [39], MEDIHEALTH adopted a similar procedure for quality control. To define and understand the problem of medication adherence, a scoping review of studies investigating factors affecting medication adherence among T2DM patients in Malaysia was conducted and summarized [40–43]. Such review provides valuable insights for the formulation of an intervention that could tackle the root cause of medication non-adherence. In addition, four T2DM Malay patients were approached to explore their related knowledge, attitude, subjective norms and perceived behavioural control towards medication adherence. Four of them were also invited to review the module and had participated in the simulation of the Program. Feedback was obtained to ensure that it fulfils the three components of cultural sensitivity as mentioned above. Nonetheless, as the studied population is Malay patients with underlying T2DM who reside in Sarawak, the content of the module will be in the Malay language and all facilitators will communicate with participants using Sarawak’s Malay dialect. As the dialect has further derived into different slangs geographically [44], such as Kuching, Samarahan, Betong and Saratok, the MEDIHEALTH program will be organized for the Malay communities who reside in Petra Jaya (Kuching Division) and Kota Samarahan (Samarahan Division) separately. The structured GBEP is conducted only once for 3 h and facilitated by one main facilitator and three assistant facilitators.

#### Facilitators

Facilitators who have experience in conducting the intervention at least five times will be eligible as facilitators for this study. Apart from training the facilitators to be familiar with and consistent in conducting the intervention, their training will also include (1) learning how to eliminate concerns and questions the patients come across pertinent to medication adherence with the mnemonic tool “ADHERE” adopted from Soto-Greene and colleagues [45]; and (2) learning patient-centred communication as advocated by Jones [46]. It encourages facilitators to be active listeners and learn how to encourage the participants to think about and understand the problems they face with medication adherence and help them decide to act. It also involves non-verbal communication skills such as nodding and making eye contact to show genuine interest in and concern about the issues raised by the participants as the intervention embraces the philosophy of patient empowerment, which had been found to be effective in engaging patients with diabetes to produce behavioural change [47, 48]. Hence, the facilitators will be trained to employ a non-didactic approach in facilitating and eliciting learning among the group members.

To ensure the consistency and correctness of the facilitators in conducting the intervention according to the intervention content and applying the communication skills learned, three sessions of the intervention prior to the actual study will be observed and assessed by the researchers on their performance in terms of coverage of all learning topics, consistency in conducting the intervention, communication with participants and responsiveness to participants’ concerns using a structured evaluation form (Additional file 3). Besides, the facilitators will also be asked to evaluate their own performance on the same aspects. They are required to achieve 90–100% of all the aspects mentioned above to be qualified as facilitators for this study.

Each session will require one main facilitator and three assistant facilitators to deliver the program. Besides, four qualified and trained facilitators are available for back-up. The number of participants for each session of the structured group-based intervention is fixed between 20 and 24 participants. Thus, during the mini-group discussion session, each facilitator will assist an average of five to six participants. Notably, all facilitators are the pharmacists who serve under the Pharmaceutical Services Division of Sarawak State Health Department. The setting of the venue for the Program is available in Additional file 4.

#### Content of the control group

Participants who are assigned to the control group will be asked to complete the questionnaire with the assistance of the facilitators. A briefing on how to answer the questionnaire will be given by the facilitators, who can answer any questions related to the questionnaire raised by the participants. However, the facilitators are not allowed to answer on behalf of the participants. After completion of the questionnaire, participants will be informed about the subsequent follow-up measurement after 1, 3, 6 and 12 months. After that, they will be dismissed and receive their usual care provided by the health clinics as before without any changes.

### Outcome measurement

The primary outcome of this study is medication adherence, whereas the secondary outcomes are the variables that contribute to the primary outcome as depicted in the conceptual framework. The details of the instrument used to measure the primary and secondary outcomes of this study are illustrated in Table 1.

**Table 1:** Outcome measurements of the study

Variables	No. of items	Measuring scale
Primary outcome		
Medication adherence [34, 35]	8	1–7 items are measured with binomial answers, which are “yes” or “no”; the last item is measured with 5-point Likert scale
Secondary outcome		
Adherence information [57]	6	5-point Likert scale
Attitude to adhere [58]	5	5-point Likert scale
Subjective norm to adhere [58]	6	5-point Likert scale
Perceived behavioural control to adhere [59]	11	5-point Likert scale
Intention to adhere [60]	3	5-point Likert scale

#### Validation of the questionnaire

All items, which are originally in English, will be translated into Bahasa Malaysia and will be back-translated into English by a group of two experienced language lecturers from the Universiti Malaysia Sarawak. Two experts in the field of behavioural studies will be invited to examine the content validity of the BM questionnaire. Furthermore, the translated questionnaire will be pre-tested among six Malay patients with T2DM from PJHC prior to the study. All comments given during the pre-test pertinent to the questionnaire design, items and ease of administration will be reported, and amendments made accordingly. The six individuals involved in the pre-test will not be included in the actual study.

#### Qualitative evaluation on effectiveness and sustainability

On the one hand, to explore how and why the program impacts the medication-taking behaviours of participants, a one-to-one semi-structured interview with five participants using purposive sampling will be conducted 1 month after the intervention. The questions that will be asked include (1) how would the GBEP MEDIHEALTH help to improve medication adherence among T2DM Malay patients; (2) how did this program help you in improving your medication adherence; (3) what is(are) the weakness(es) of the program and what could be done to improve it; and (4) would you recommend this SGBI to other T2DM Malay patients and why.

On the other hand, to explore sustainability in terms of organizational routines and institutional standards [22], two main facilitators and two managerial officers of Sarawak Pharmaceutical Services Division who oversee the implementation of the program will be interviewed after 12 months of the intervention. The aspects of sustainability of the program that will be discussed include (1) how would the department sustain the manpower required in implementing the program; (2) how would the department sustain the long-term implementation of the program based on the cost involved in running the program; (3) how and why would the program gain support from top management; (4) how and why would the program be implemented in other facilities; and (5) how and why would the program be implemented regularly. All interviews will be audio-recorded and transcribed verbatim for further analyses.

#### Treatment fidelity

Treatment fidelity of the structured GBEP will be evaluated using the concept and strategies developed by the Treatment Fidelity Workgroup of the National Institutes of Health Behaviour Change Consortium [49]. The framework of treatment fidelity strategies for this study is depicted in Table 2.

**Table 2:** Framework of treatment fidelity strategies

Components	Goal	Strategies
Study design	Ensure the same treatment dose within conditions and equivalent dose across conditions.	1. The structured GBEP is designed to be completed within 3 h with an allowance of 15-min deviation. 2. The intervention manual will ensure all facilitators conduct the intervention in a consistent manner. 3. Observation on 3 sessions of the intervention conducted by the involved facilitators prior to the actual study will be done by the researchers to assess the consistency and appropriateness in conducting the intervention. Feedback will be given to the facilitators by the observers after the observation. The facilitators will also discuss the issues faced during the intervention with the researchers. 4. All facilitators are acquired to adhere to the time allocated for each activity throughout the intervention.
	Plan for implementation setbacks.	Have an extra 4 qualified and trained facilitators in case of unavailability of the involved facilitators.
Provider training	Standardize training.	All the qualified and involved facilitators together with the 4 back-up facilitators will be trained together to ensure consistency in conducting the intervention. Observation on 3 sessions of the intervention conducted by the involved facilitators prior to the actual study will be able to ensure the actual performance of the involved facilitators.
	Ensure provider skill acquisition.	A scoring scale to assess the qualification and consistency of the facilitators in conducting the intervention will be practised.
	Minimize “drift” in provider skills.	During the actual study, the researchers will still observe the intervention conducted by the facilitators to ensure the consistency of the intervention. Should the researchers observe below 90% of consistency as compared to the training sessions, the reasons that caused the inconsistency will be investigated and reported.
	Accommodate provider differences.	All facilitators are pharmacists who work in the Pharmaceutical Services Division, Sarawak State Health Department. Hence, the facilitators have a similar pattern of knowledge background and are considered expert related to the study.
Treatment delivery	Control for provider differences.	The facilitators have similar background and have the same training at the same time.
	Reduce differences within treatment.	A scripted intervention manual is available in the form of Microsoft PowerPoint slides and used by the facilitators.
	Ensure adherence to the treatment protocol.	During the actual study, the researchers will still observe the intervention conducted by the facilitators and will be video-recorded to ensure the consistency of the intervention. Should the researchers observe below 90% of consistency as compared to the training sessions, the reasons that caused the inconsistency will be investigated and reported.
	Minimize contamination between conditions.	This is a randomized controlled trial with blinding on the researchers, facilitators and participants to treatment allocation prior to the intervention.
Treatment receipt	Ensure participant comprehension.	1. Participant understanding on the message will be evaluated with the scales developed to measure the impact of the intervention on the psychosocial variables of the participants. A comparison between the intervention group and the control group will show whether the improvement in the psychosocial variables is due to chance or is because of the intervention. 2. A qualitative interview after the intervention will enable the researchers to know how the intervention impacts their medication-taking behaviour.
	Ensure participant ability to use cognitive skills.	1. The facilitators work with the participants until they can demonstrate correct medication-taking skills. 2. Hypothetical situations that the participants may face in real life will be addressed during group discussion and sharing on their reasons for non-adherence and the method that they will adopt to overcome the problem.
	Ensure participant ability to perform behavioural skills.	The facilitators work with the participants until they can demonstrate correct medication-taking skills.
Enactment of treatment skills	Ensure participant use of cognitive skills.	The use of a medication chart prepared by the participants will show how well they comprehend the medication-taking skills.
	Ensure participant use of behavioural skills	Medication adherence will be measured after 1, 3 and 6 months of the intervention to ensure that the messages conveyed through the intervention are translated into action and such action is maintained.

### Data analysis

All data recorded will be scrutinized for accuracy and completeness. Data obtained will be entered into an IBM SPSS data sheet. Data collected will be explored, checked and cleaned to detect any missing data or wrong data entries. Data will later be entered and stored in password-protected electronic storage. Original documents, including signed informed consent and completed questionnaires, will be retained by the principal investigator for a minimum of 15 years. All study documents will be stored in locked cabinets and electronic data encrypted with restricted access. Enumerators will employ multiple attempts to follow up all the participants to minimize missing outcome data. Firstly, the enumerators will check the completeness of the questionnaires upon their submission during follow-up visits. If the participants do not show up during the follow-up visits, they will be contacted and interviewed via a phone call. If they cannot be contacted via a phone call, the last resolution will be a home visit. However, should a missing outcome data become unavoidable, multiple imputation analysis using Stata MI commands with a memory-efficient style (mlong) and five imputations will be employed [50].

The overall mean will be computed using IBM SPSS software for outcome variables: adherence information, attitude to adhere, subjective norm to adhere, perceived behavioural control to adhere and intention to adhere. The outcome variable medication adherence will be categorized into two: adherence and non-adherence, before the researcher proceeds with univariate and multivariate analyses. For the outcome variable medication adherence, a MMAS-8 score >6 will be categorized as adherence, whereas a MMAS-8 score <6 will be categorized as non-adherence.

Assumption checking will be done to decide whether a parametric test or non-parametric test is to be employed in the data analysis. The independent *t* test or Mann-Whitney *U* test will be used to examine the difference in medication adherence levels and psychosocial variables among participants between the intervention and control groups. To test the change in secondary outcome variables before and after 1, 3, 6 and 12 months of the intervention, repeated-measures ANOVA using IBM SPSS software will be used to assess the difference in the between-groups effect (inter-group comparison) and within-subject effect (time effect). Repeated measures ANOVA is equivalent to ANOVA for related samples and is the extension of the paired *t* test. Multivariate analysis will be used in this study to eliminate confounding effect and prevent type 1 error inflation. For the testing of hypotheses, path analysis using a generalized estimating equation (GEE) with the Stata Version 14 [51] xtgee command will be conducted. The GEE procedure is an extension of the generalized linear model for analysis of repeated measurements. The path coefficients will be obtained from the *β* weights of the GEE analyses. All statistical analyses will be two-sided with *p* < 5% for consideration of statistical significance.

For the qualitative data collected through the semi-structured interview, the constant comparative method for qualitative analysis [52] with the assistance of ATLAS.ti 5 [53] will be used to identify the themes related to the questions.

## Discussion

While previous studies have provided little empirical evidence of culturally sensitive health-promoting interventions [38], only tested on specific minority populations [54], this study aims to provide evidence on the effectiveness of a structured GBEP that employs psychosocial and behavioural approaches, with multiple theoretical grounding and a culturally sensitive approach in promoting medication adherence among Malay patients with underlying T2DM. Nevertheless, the testing of the integrated model (TPB and IMB) in explaining medication adherence is also contributing to the extant body of knowledge. Moore and Evans argued that it is important to take the context into consideration when choosing the theory that “best fits” the complex population being tested [55]. Therefore, we decided to test the two theories—TPB and IMB—among our Malay patients with T2DM. These theories have been applied to the testing of medication adherence and proven effective among patients with T2DM [24, 25]. Nonetheless, the variables of the TPB and IMB were found to include the factors that influence medication adherence among Malay T2DM patients as revealed by the scoping review and interview with key informants as mentioned before. Hence, the findings of this study could enrich these existing theories through their application to a multicultural Malaysian context.

For healthcare professionals, the quantitative and qualitative findings of this study could assist in identifying the possible shortfall of the program and provide a baseline for future studies. Notably, we employ both inductive and deductive reasoning on the mechanism of the program because we agree with Astbury and Leeuw that “program theory building with mechanisms involves constant shuttling between theory and empirical data, using both inductive and deductive reasoning” because the mechanisms “are usually hidden” and “sensitive to variations in context” [56].

Lastly, the findings on the effectiveness of the GBEP in improving medication adherence could become evidence for policymakers and authorities in justifying the resources spent in running the program. Furthermore, this one-off, pharmacist-led and multiple-theoretical-grounding structured GBEP module could be expanded to other types of medication management, which could complement the existing DMTAC in improving medication adherence. It is expected that the combination of both individual and group-based approaches could maximize the coverage of patients who require special care in their medication management and reduce the disease burden due to medication non-adherence.

## Trial status

This protocol is registered with ClinicalTrials.gov with trial identifier NCT03228706. It was first posted on July 25, 2017, and was updated on January 25, 2018 (version 1.3; https://clinicaltrials.gov/ct2/show/NCT03228706). Recruitment began on August 1, 2017, and is expected to be completed on July 31, 2018.

## Additional files

Additional file 1: SPIRIT fillable checklist. (PDF 179 kb)

Additional file 2: MEDIHEALTH content and intervention mapping. (PDF 193 kb)

Additional file 3: Facilitator competency and inter-coder reliability assessment form. (PDF 300 kb)

Additional file 4: Setting of the venue for the MEDIHEALTH program. (PDF 326 kb)

## Abbreviations

GBEP: Group-based educational program
HIV: Human immunodeficiency virus
IMB: Information-motivation-behavioural skills model
KSHC: Kota Samarahan Health Clinic
KYM: Know Your Medicine
MEDIHEALTH: Know Your Medicine – Take it for Health
MMAS-8: 8-item Morisky Medication Adherence Scale, Malaysian specific
MOH: Ministry of Health, Malaysia
DMTAC: Diabetes Medication Therapy Adherence Clinic
NHMS: National health morbidity survey
PJHC: Petra Jaya Health Clinic
PSD: Pharmaceutical Services Division
T2DM: Type 2 diabetes mellitus
TPB: Theory of planned behaviour
