# Correction to: Effectiveness and sustainability of a structured group-based educational program (MEDIHEALTH) in improving medication adherence among Malay patients with underlying type 2 diabetes mellitus in Sarawak State of Malaysia: study protocol of a randomized controlled trial

**DOI:** 10.1186/s13063-019-3348-x

**Published:** 2019-05-10

**Authors:** Chuo Yew Ting, Shahren Ahmad Zaidi Adruce, Mohamed Azmi Hassali, Hiram Ting, Chien Joo Lim, Rachel Sing-Kiat Ting, Abu Hassan Alshaari Abd Jabar, Nor Anizah Osman, Izzul Syazwan Shuib, Shing Chyi Loo, Sui Theng Sim, Su Ee Lim, Donald E. Morisky

**Affiliations:** 1 Institute of Borneo Studies, Universiti Malaysia Sarawak, Kota Samarahan, Sarawak Malaysia; 2 Discipline of Social and Administrative Pharmacy, School of Pharmaceutical Sciences, Universiti Sains Malaysia, Penang, Malaysia; 3 Sarawak Research Society, Sarawak, Malaysia; 4 Clinical Research Center, Sarawak General Hospital, Sarawak, Malaysia; 5 Jeffrey Cheah School of Medicine and Health Sciences, Monash University Malaysia, Selangor, Malaysia; 6 Pharmaceutical Services Division, Sarawak State Health Department, Sarawak, Malaysia; 7 Pharmacy Practice and Development Division, Sarawak State Health Department, Sarawak, Malaysia; 8 Pharmacy Enforcement Division, Sarawak State Health Department, Sarawak, Malaysia; 9 Department of Community Health Sciences, UCLA Fielding School of Public Health, Los Angeles, CA USA

**Correction to: Trial**

**https://doi.org/10.1186/s13063-018-2649-9**

After publication of the original article [1], the authors have notified us that there are changes to the primary outcome of the study, instrument, subject’s inclusion criteria, the funding and acknowledgements. These changes were made during the recruitment of participants and after approved by the Medical Research and Ethics Committee (MREC), National Institutes of Health Malaysia, on 16th November 2018.

1. We added HbA1c level as primary outcome, where we hypothesized the improvement in medication adherence will improve the HbA1c level. Hence, the revised conceptual framework of the study (Fig. 1) is shown on page 2.

**Fig. 1:**
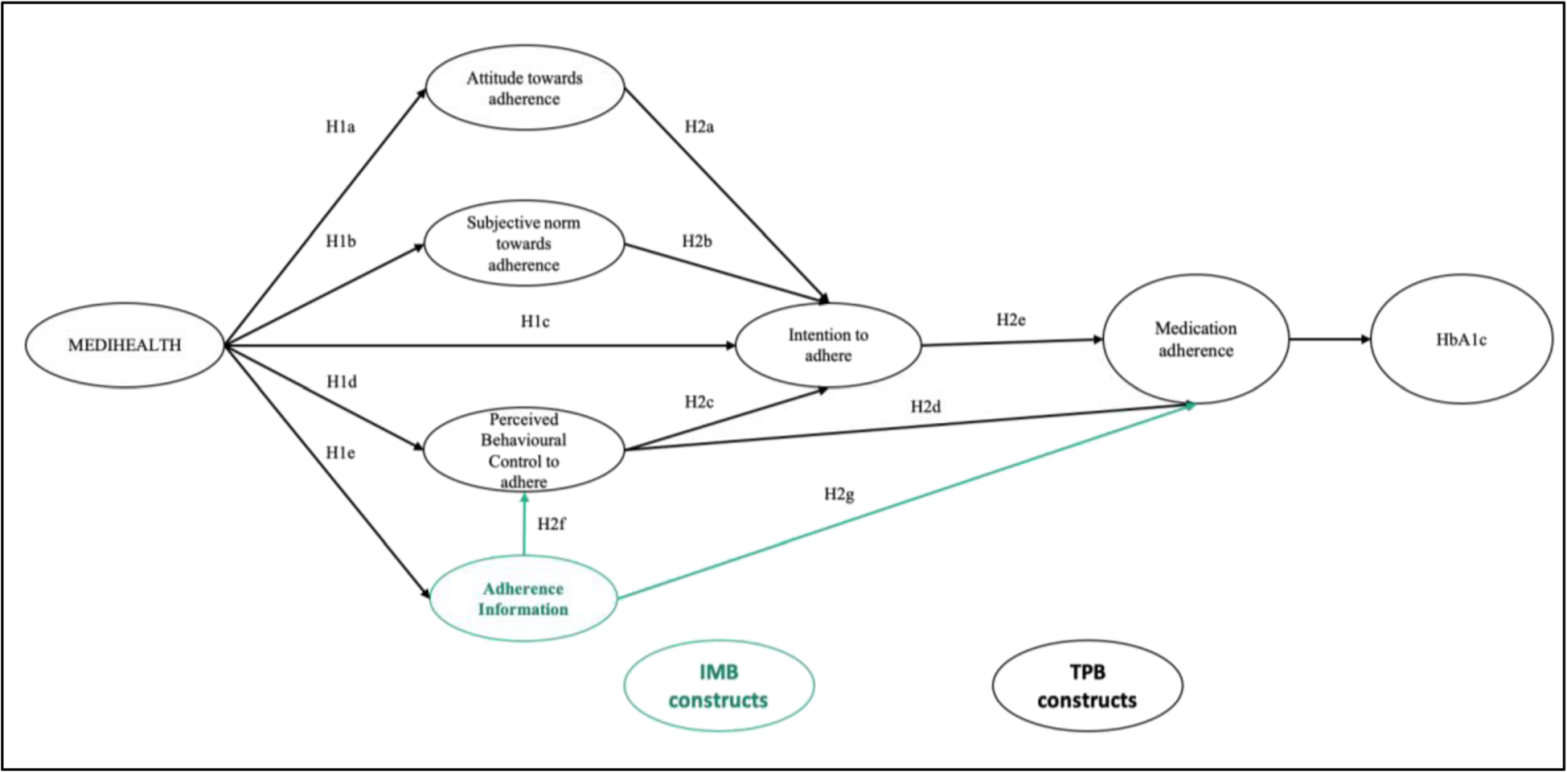
Conceptual framework of the study (TPB constructs are in black while IMB construct is in green)
2. We replaced the use of MMAS-8 with the self-efficacy for appropriate medication use scale (SEAMS) [2]. This change is made throughout the article, tables, figures and supplementary materials. We sought a validated subjective measurement on the level of patients’ self-efficacy in adhering with the dosing regimen during implementation phase of medication adherence. Such subjective measurement must be validated among T2DM patients or at least chronic diseases. Nonetheless, most of the T2DM patients who have poor medication adherence were elderly and had relatively lower literacy due to less education opportunity in the past. Hence, the SEAMS which had been validated among low-literacy patients with chronic disease (patients with coronary heart diseases and other comorbidities) was the best-suited measurement for this study. SEAMS has good internal consistency (Cronbach’s alpha = 0.89) and could explain 52.3% of the scale’s variance. The answers to all the questions are on 3-point scales, with 1 indicating “not confident”; 2 indicates “somewhat confident”; and 3 indicates “very confident”. The total scores of the scale range from 13 to 39, where a higher score means greater adherence level. Notably, SEAMS has no cut-off point to categorize the level of medication adherence. Hence, we took the mean (26) of the score range (13–39), as cut-off point for poor adherence (total score < 26) and good adherence (total score ≥ 26).
3. We changed the inclusion criteria to: (1) Malay Type 2 Diabetes Mellitus patients, (2) HbA1c level was greater than 7% and the (3) SEAMS total score was less than 26 upon recruitment.
4. The funder no longer provides financial support for the use of the MMAS-8.
5. We add the following to acknowledgments, “We thank the Dr Jessica Corwin (MD.), from TPMG Northern California for the permission to translate and to use the Self-efficacy for Appropriate Medication Use Scale (SEAMS).” We exclude the following from the acknowledgements, “The MMAS-8 content, name and trademarks are protected by US copyright and trademark laws. Permission to use the scale and its coding is required. A license agreement is available from Donald E. Morisky, ScD, ScM, MSPH, 14725 NE 20th St Bellevue, WA 98007, USA (dmorisky@gmail.com).”

This erratum also corrects the authorship of this article. The correct author list is as follows: Chuo Yew Ting, Shahren Ahmad Zaidi Adruce, Mohamed Azmi Hassali, Hiram Ting, Chien Joo Lim, Rachel Sing-Kiat Ting, Abu Hassan Alshaari Abd Jabar, Nor Anizah Osman, Izzul Syazwan Shuib, Shing Chyi Loo, Sui Theng Sim and Su Ee Lim.
