# Supplementary material for: Effectiveness and sustainability of a structured group-based educational program (MEDIHEALTH) in improving medication adherence among Malay patients with underlying type 2 diabetes mellitus in Sarawak State of Malaysia: study protocol of a randomized controlled trial
Source: Trials. 2018 Jun 5;19:310. doi: 10.1186/s13063-018-2649-9 (PMC5989376; doi:10.1186/s13063-018-2649-9)
Supplement: Supplementary file 2 — MEDIHEALTH content and intervention mapping. (PDF 193 kb) [file 13063_2018_2649_MOESM2_ESM.pdf]

MEDIHEALTH Program

Introduction

Amidst the high disease burden, non-adherence to medications among Type 2 Diabetes Mellitus (T2DM) patients had reported to be gnarly and devastating. Sarawak Pharmaceutical Services Division had formulated a pharmacist-led, multiple theoretical grounding, culturally sensitive and structured group-based program, namely "Know Your Medicine – Take if for Health" (MEDIHEALTH), to improve medication adherence among T2DM patients.

Objective

To improve the medication adherence among T2DM patients through enhancing their attitude, subjective norms, self-efficacy, skills, motivation, knowledge about the disease and medication, as well as addressing the common wrong perception about the medicines such as side effects, inferiority of generic medicines, misuse of traditional and complementary medicines.

Target audiences

T2DM patients who has problems to adhere with their medication both unintentionally or intentionally (Referred by Physician/Pharmacist). Notably, since this program emphasizes on cultural sensitivity, T2DM patients with same ethnicity and speak same native language will be grouped together.

Language

The content of the program is available in Bahasa Malaysia, English and Mandarin. As aforementioned that this program emphasizes on cultural sensitivity, the facilitator will be communicating with the participants in native language that suits the audiences with same ethnicity and have same native language. For example, if the audiences are Iban T2DM patients, the content of the program will be in Bahasa Malaysia, while the facilitators who can speak Iban language will be facilitating the program.

Number of participants

12-24 per groups

Duration

3 hours, including 35 minutes of answering survey questionnaire before and after the program.

Content of the program:

The intervention mapping of MEDIHEALTH program is showed in Figure 1. The details of the program are available at Table 1.

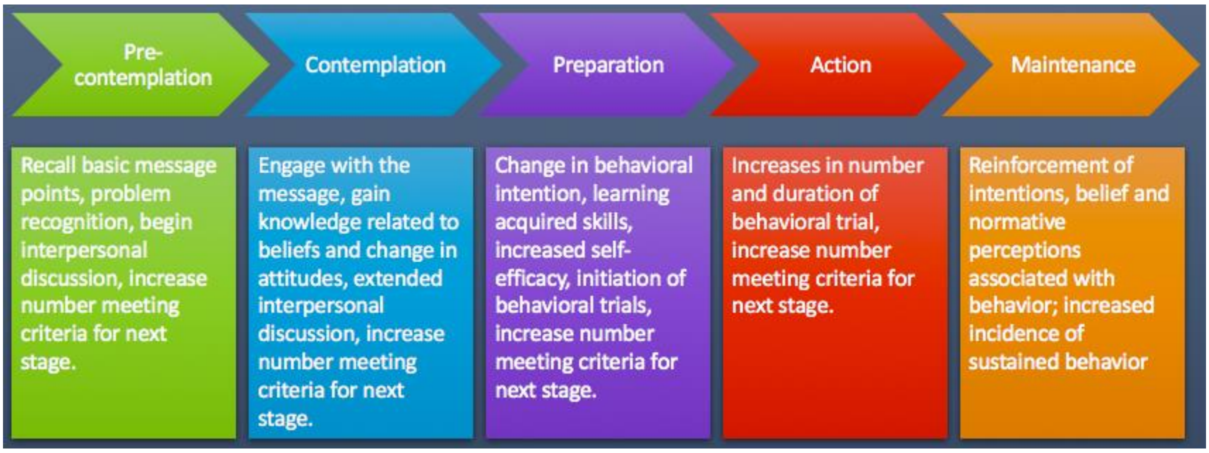

Figure 1: Intervention mapping based on Transtheoretical Model (Prochaska & Velicer 1997)

Source: Slater MD. Integrating application of media effects, persuasion, and behavior change theories to communication campaigns: A stages-of-change framework. Health Communication. 1999 Oct 1;11(4):335-54.

Table 1: Contents of the MEDIHEALTH (Copyright of Pharmaceutical Services Division, Sarawak State Health Department)

Stage of Change	Change techniques	Theoretical support	Content of intervention	Times Allocated
Pre-contemplation	Recall basic message points, problem recognition, begin interpersonal discussion	(1) Social Learning Theory<br><br>(2) Elaboration-Likelihood-Model	Pre-test	15 minutes
			Breaking respondents into 4 groups (5-6 persons/group) and assign group leader of each group before the program begin. Main facilitator welcome the participants and introduce the other facilitators. Group members have ice-breaking session to introduce themselves to one another by telling their name, their residential area, their favorite food and how long they had been diagnosed with T2DM.	25 minutes
			Explain the prevalence and complications of T2DM disease globally and locally.	5 minutes
			A welcoming speech by Deputy Director in Pharmacy Program of Sarawak State Health Department and to explain the rationale of conducting the program and the expected outcome of the program.	5 minutes
Contemplation	Gain knowledge about the belief and change in attitude	(1) Theory of planned behavior<br><br>(2) Information-Motivation-Behavioral Skill model	Group members share their attitude and belief on taking medications through writing it down on a mahjong paper with assistance of facilitators. Only key words are written.	10 minutes
			Facilitators will go through the answers given by respondents and recognize the attitude and belief on medication taking behaviors that are correct and rectify those that are incorrect.	25 minutes
			A role play involving all participants to illustrate how the T2DM happen, how the medicines work and the complications of the disease if they did not adhere with their medications	20 minutes

Preparation	Change in behavioral intention, learning skill, increase self-efficacy	(1) Self-efficacy Theory	Address the physical barriers that cause unintentional medication non-adherence such as no time or no transport to collect medicines, forgetfulness, unable to understand the medication label and any issues raised by participants	10 minutes
5 minutes' break for participants. At the same time facilitators will distribute the medications belonged to the participants that had been dispensed by outpatient pharmacy department of HCPJ/HCKS.				
Action	Act on the message, increase number and duration of behavioral trial	(1) Information-motivation-behavioral skill model<br><br>(2) Self-efficacy Theory	Main facilitator will brief participants on the use of the medication chart and they will be asked to write down all the medications they have on the chart with the dosage regimen. Subsequently, they need to identify the types of medications which have problem to adhere and discuss with the group on the method they want to take to improve it.	15 minutes
			Simulation of taking medications according to the medication chart using their own medications one by one with observation by facilitators to ensure the correctness. The simulation only acquires the participants to show the medicines they are going to take and tell the quantity and timing they need to take the medicines to the group facilitator. Facilitators will be able to evaluate the actual understanding and skill in taking medications of participants and rectify right away. Repeat testing the skill of participants until they can demonstrate correct medication taking skill on all their medications.	10 minutes
Maintenance	Reinforce intention, belief and normative perceptions towards behavior	(1) Elaboration likelihood Model<br><br>(2) Self-efficacy Theory<br><br>(3) Social Learning Theory	Present a 5 minutes' video clip that show the message delivered by the officer in charge of Sarawak Traditional and Complementary Medicine Division about the importance of taking medication as prescribed amidst their traditional medicines' consumption.	5 minutes
			Group members share their learnings to each other and main facilitator recapitulate the important message of this program.	10 minutes
			Post-test	20 minutes
			Total Time	180 minutes
