# Supplementary material for: Effectiveness and sustainability of a structured group-based educational program (MEDIHEALTH) in improving medication adherence among Malay patients with underlying type 2 diabetes mellitus in Sarawak State of Malaysia: study protocol of a randomized controlled trial
Source: Trials. 2018 Jun 5;19:310. doi: 10.1186/s13063-018-2649-9 (PMC5989376; doi:10.1186/s13063-018-2649-9)
Supplement: Supplementary file 3 — Facilitator competency and inter-coder reliability assessment form. (PDF 300 kb) [file 13063_2018_2649_MOESM3_ESM.pdf]

Facilitator Competency and Inter-Coder Reliability Assessment Report (Appendix F)

Assessing researcher: (1) ___________________________ (2) ______________________________(3)________________________________

Assessed Main facilitator: _________________________________

Assistant facilitators: (1)_____________________________(2)______________________________(3)________________________________

Date: ______________________________________

Venue: _____________________________________

Number of participants: ________________________

Table 1. Coverage of messages delivery strategies by facilitators

**Stage of Change**	**Message delivery strategies (please tick in the if it is covered)**	**Times Allocated (Min)**	**Time Used (Min)**	***Discrepa -ncy (Min)**
Pre-contemplation	Set up the venue before the program begin<br>Registration of participants	Preset		
	Pre-test	15		
	Ice breaking 1:Nametag	15		
	Ice breaking 2 with subsequent group assignment: Sociogram	10		
	Prevalence of T2DM and general medication adherence problems	5		
	“Harapan Anda”	5		
	Welcoming video by Deputy State Health Director (Pharmacy) dan Ustaz Mohd Ihsan	5		
Contemplation	Group members sharing (World Café) on their attitude and belief on taking medications through writing it down on a mahjong paper with assistance of facilitators. Four questions will be asked:<br>(1) What is Type 2 Diabetes Mellitus (T2DM)?<br>(2) What is the symptoms of T2DM?<br>(3) What is the treatment for T2DM?<br>(4) What are the myths and facts about medications for T2DM?	10		

Facilitator Competency and Inter-Coder Reliability Assessment Report (Appendix F)

	Facilitator go through the first question, “what is the T2DM?”	5		
	Facilitator go through the second question, “what is the symptoms of T2DM?	5		
	Facilitator go through the third question, “what is the treatment for T2DM?”	20		
	15 minutes’ break for participants. At the same time facilitators will distribute the medications belonged to the participants that had been dispensed by outpatient pharmacy department of HCPJ/HCKS.			
	Facilitator go through the forth question, “Myths and Facts about medication for T2DM”	15		
Preparation	Introduction of value-added services provided by government to address transportation problem and inconveniences faced by patients to obtain continuous medicine supply	5		
	How to read the medicine label and the proper way of keeping medicines	5		
	Brief participants on the use of the medication chart<br>Ask participants to write down all the medications they have on the chart with the dosage regimen.<br>Ask participants to identify the types of medications which have problem to adhere and discuss with the group on the method they want to take to improve it.	15		
Action	Simulation of taking medicines according to the medication chart prepared	10		
Maintenance	Present video clips that show the message delivered by (1) Dr in charge of Sarawak Traditional and Complementary Medicine Division about the importance of taking medication as prescribed and (2) Religion leader Ustaz Mohd Ihsan	5		
	Tree of knowledge	10		
	Post test	20		
	Total time taken	180		

* Total discrepancies must be less than 90%

Facilitator Competency and Inter-Coder Reliability Assessment Report (Appendix F)

Facilitator Competency Assessment

Name of facilitator: ______________________________________(Main / Assistant)

Assessing researcher: (1) __________________(2) _________________(3)_________________

Table 2. Facilitator Competency Assessment (Gottleib 2000; Soto 2004; Jones 2004)

**Criteria****	**Comments (Yes/No with justification)**
(1) The facilitator acknowledges the concerns of patients.	
(2) The facilitator discusses with patients on their concerns.	
(3) The facilitator **h**andles concerns of patients promptly.	
(4) The facilitator assesses patients' health literacy and identify possible barriers to adherence.	
(5) The facilitator suggests appropriate and feasible solution to the patient.	
(6) The facilitator elicits patients' willingness to adhere with medications for long-term.	
(7) The facilitator adopts friendly attitude and not business-like attitude	
(8) The facilitator avoids medical jargon	
(9) The facilitator uses short sentences	
(10) The facilitator gives clear instruction and repeat the instructions	
(11) The facilitator gives specific and detailed information	
(12) The facilitator ensures the patients understand by asking them to repeat what had been said	
(13) The facilitator always show genuine concern through nonverbal communication (eye contact and nodding head)	

** Must fulfil at least 90% of the criteria

Facilitator Competency and Inter-Coder Reliability Assessment Report (Appendix F)

Consistency of Main/Assistant Facilitators

Name of facilitator: ___________________________________(Main / Assistant)

Assessing researcher: (1) ________________(2) _______________(3)_______________

Based on **previous three (3) sessions of the program** conducted by the main facilitator together with the assistant facilitators, please evaluate their consistency on time control and competency.

Total time taken to conduct the program:

1st session: _______________ minutes

2nd session: _______________ minutes

3rd session: _______________ minutes

Table 3. Consistency of main/assistant facilitators

Criteria	Comments (Yes/No with justification)	Percentage of discrepancy***
Time taken to conduct each session of the program is within the allowance		
Time taken to conduct each session of the program is consistent		
The competency of the main/assistant facilitator is consistent (Based on the Facilitator Competency Assessment)		

*** Discrepancy on time of each session must be less than 10%

Comments by researcher:

Shortfall(s):
Recommendation(s):

Facilitator Competency and Inter-Coder Reliability Assessment Report (Appendix F)

Inter-coder Reliability Assessment

Table 4. Comparison on the evaluation of different researchers on the competency and consistency of facilitators

	**Part A:** Evaluation on coverage of message delivery strategies	**Part B:** Evaluation on time used to cover each part of message delivery strategies	**Part C:** Evaluation on total discrepancy on time	**Part D:** Evaluation on facilitator competency assessment	**Part E:** Evaluation on consistency of facilitators
1st Researcher					
2nd Researcher					
3rd Researcher					
****Discrepancies					

**** Total discrepancies should be less than 10%

Name of researchers who evaluate the facilitators' competency and consistency:

(1) ______________________________________

(2) ______________________________________

(3) ______________________________________

Inter-Coder Reliability Result: __________________% (Not less than 90%)

Facilitator Competency and Inter-Coder Reliability Assessment Report (Appendix F)

Table 5. Discussion on the aspects that cause the inter-coder reliability to be less than 90% (if applicable):

Discrepancies on Part A/B/C/D/E	Discussion	Agreement after discussion

Final Inter-Coder Reliability Result: _________________% (Not less than 90%)
