# Supplementary material for: Effectiveness and sustainability of a structured group-based educational program (MEDIHEALTH) in improving medication adherence among Malay patients with underlying type 2 diabetes mellitus in Sarawak State of Malaysia: study protocol of a randomized controlled trial
Source: Trials. 2018 Jun 5;19:310. doi: 10.1186/s13063-018-2649-9 (PMC5989376; doi:10.1186/s13063-018-2649-9)
Supplement: Supplementary file 4 — Setting of the venue for the MEDIHEALTH program. (PDF 326 kb) [file 13063_2018_2649_MOESM4_ESM.pdf]

Setting of the venue for MEDIHEALTH program

The setting of the venue for intervention group will be in 4 mini groups (maximum 24 participants). The participants will be assigned to 4 mini groups during the ice-breaking session of the intervention. The setting is illustrated as below:

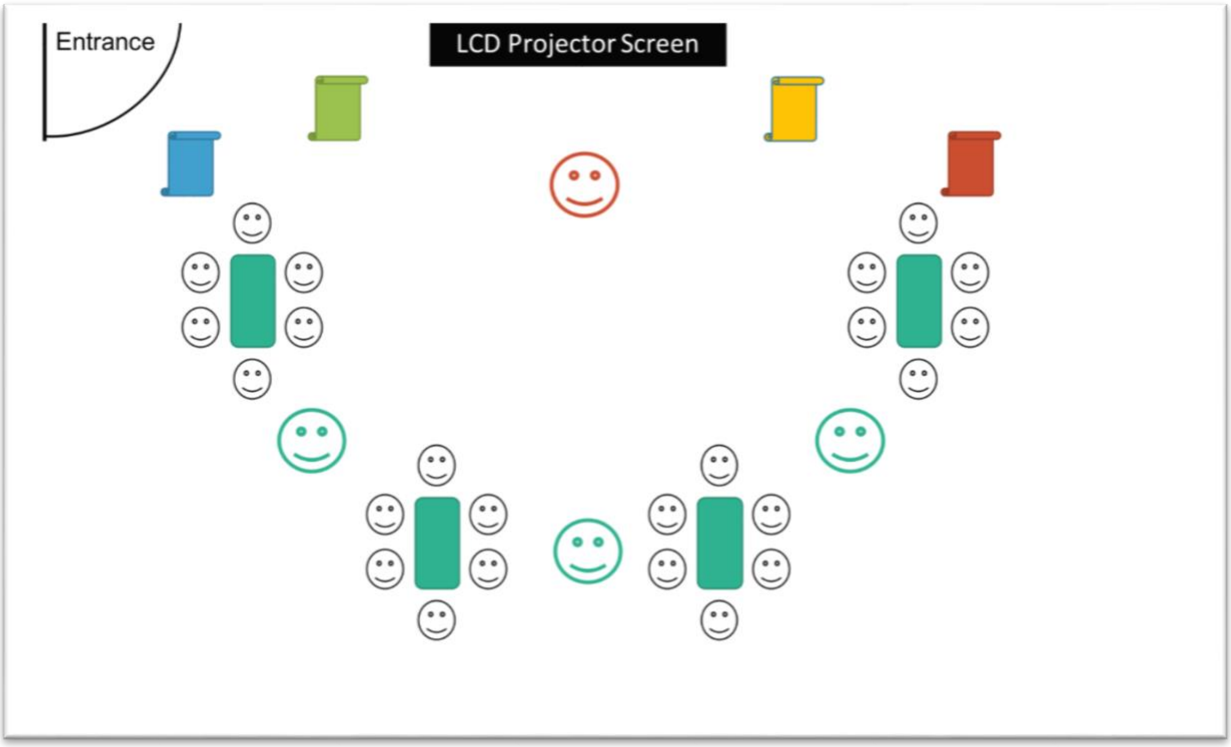

☺ = Participant; ☺ = main facilitator; ☺ = assistant facilitator; Green rectangle = table

= Banner that contain message to promote the attitude towards medication adherence (Fig. 1)

= Banner that contain message to promote the subjective norms towards medication adherence (Fig. 2)

= Banner that contain message to promote the perceived behavioural control towards medication adherence (Fig. 3)

= Banner that contain message to promote the intention to adhere with medication (Fig. 4)

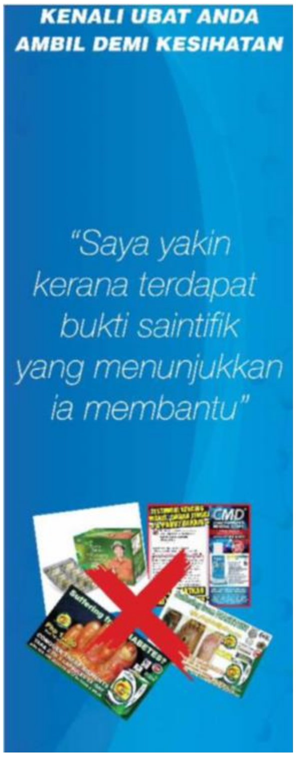

Fig. 1 Know your medicine-Take it for health. "I am confident because there are scientific evidences which prove it helps"

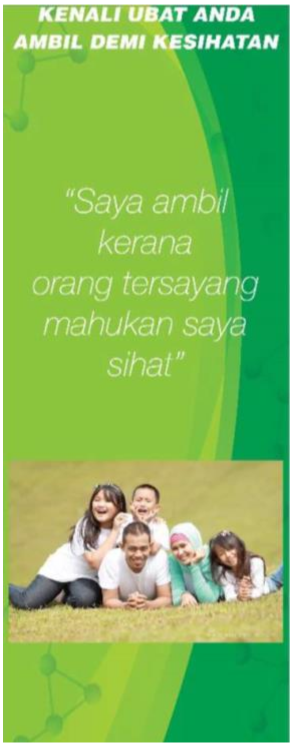

Fig. 2 Know your medicine-Take it for health. "I take because people that I love want me to be healthy"

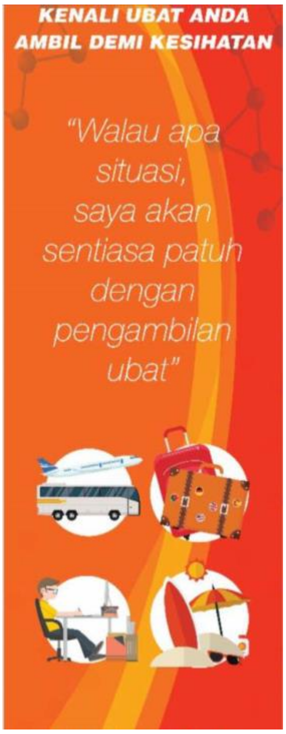

Fig. 3 Know your medicine – Take it for health. "No matter what the situation, I will continue to adhere with medication"

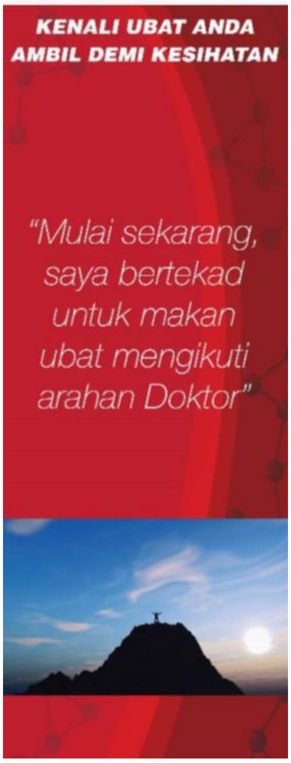

Fig. 4 Know your medicine – Take it for health. "From now onwards, I decide to take medicines as instructed by doctor"

On the other hand, the setting of the venue for control group will be in classroom setting as illustrated below (only applicable to Program conducted during the study period):

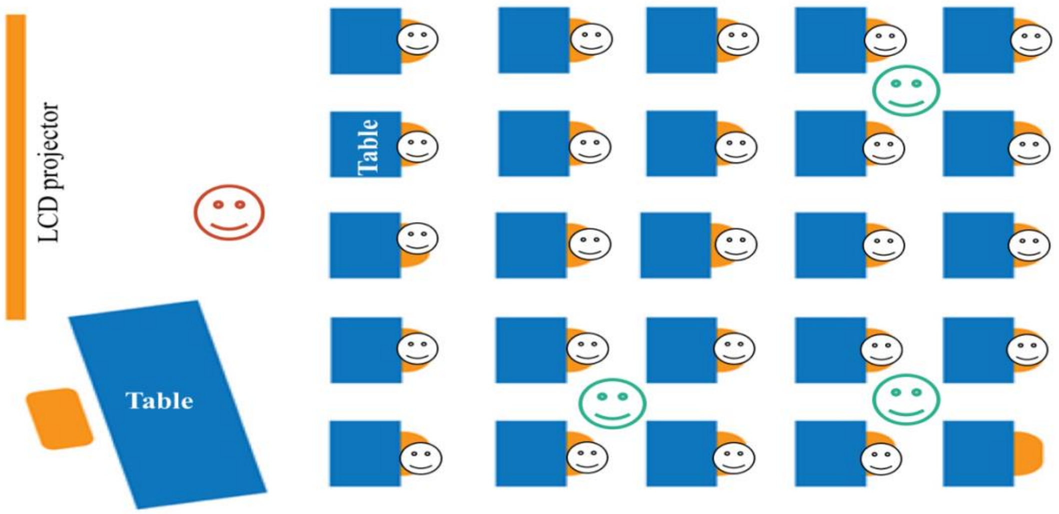

☺ = Participant; ☺ = main facilitator; ☺ = assistant facilitator
